# Adapting the *buying funnel* model of consumer behavior to the design of an online health research recruitment tool

**DOI:** 10.1017/cts.2017.17

**Published:** 2017-08-14

**Authors:** Aalap Doshi, Lisa Connally, Meghan Spiroff, Anita Johnson, George A. Mashour

**Affiliations:** 1 Michigan Institute for Clinical and Health Research, University of Michigan, Ann Arbor, MI, USA; 2 Office of Research, University of Michigan, Ann Arbor, MI, USA

**Keywords:** Health research recruitment, research recruitment registry, shopping funnel, consumer behavior, user experience

## Abstract

**Introduction:**

UMHealthResearch is the University of Michigan’s digital health research recruitment platform. It allows health researchers to connect efficiently with potentially eligible volunteers.

**Methods:**

In 2013, the UMHealthResearch team strategically adapted a consumer behavior model, the buying funnel, to create the Digital Health Research Participation Funnel. The Digital Health Research Participation Funnel was then used to design a more active way for potential participants to volunteer for research studies through UMHealthResearch.

**Results:**

In the 5 years before the redesign (2007–2012), an average of 1844 new accounts were created every year, whereas in the completed years after the redesign (2013–2016) the annual average improved to 3906, an increase of 111%.

**Conclusion:**

Although a randomized design was not possible in this instance, these preintervention and postintervention data suggest that the focus on user experience is an effective strategy for improving web-based research recruitment platforms.

## Introduction

Recruiting appropriate study subjects is critical for conducting sound research to improve health and medical care. Yet, enrolling volunteers into health research remains a significant challenge, as many teams do not have access to systems that facilitate efficient and effective recruitment from specific healthy and patient populations. Typically, health research opportunities tend to be broadly dispersed through a variety of channels. This creates a difficult and inefficient environment for patients and community members to proactively find studies in which they can be involved. To overcome this barrier, the Michigan Institute for Clinical & Health Research, a translational science institute funded by the National Institutes of Health, at the University of Michigan developed UMHealthResearch.org (formerly UMClinicalStudies.org), a web application that links the community to a single gateway for health research opportunities [1].

Before 2013, UMHealthResearch was only designed to be a registry in which volunteers could sign up to receive potential study matches at a later date. This signified a passive approach to volunteering for health research, in which someone would register and wait to be contacted by research teams. We hypothesized that shifting from passive to active engagement would result in higher registration. In this report, we detail our model and process to update UMHealthResearch to provide potential volunteers with a more active way to signify interest and get involved in specific health research opportunities that fit their interests.

Providing opportunities for active research volunteering is important for potential volunteers, as people’s motivation to participate in research is often fleeting. For potential research volunteers, their goal is to sign up for specific research studies being conducted that fit their needs and interests. However, the former registry version of UMHealthResearch did not provide a timely or straightforward method for volunteers to actively show interest in specific research. Thus, it did not capitalize on their switch from deliberating about participation to implementing their goal of participating.

In many ways, making decisions about participating in research is similar to the process of shopping for a product or service. For example, volunteers seek to sign up for specific studies that seem most suited to their needs, just as shoppers seek to purchase a product or service that fits their needs. Thus, models of consumer behavior, such as the buying funnel, are highly relevant and easily adapted to help us consider potential volunteers’ thoughts and behaviors when recruiting for research studies.

### The Buying Funnel

The buying funnel (also known as a shopper’s funnel, sales funnel, or sales cycle) is a staged process that a consumer goes through in order to purchase a product or service [2]. This model posits that consumers pass through four stages of cognition and action as they decide whether and what product or service to purchase [3]. Specifically, consumers (1) become aware of products or services, (2) research their options, (3) make a decision, and (4) purchase one of the options.

In his work consulting with businesses through Google Ventures, Michael Margolis has adapted the buying funnel to be a user experience design tool for online stores [4]. He proposes 6 general stages in this funnel for Web site design to enhance the user experience when purchasing a service or product:

1. *Discover: gather options and establish criteria*—in this stage, customers try to determine what products are available, what their requirements and criteria are for the product they seek, and which sites are credible sources for information about the product. Therefore, in this stage, Web sites must establish trust with their users and provide clear descriptions of products and their features.
2. *Select: make a short list*—in this stage, customers choose a set of product options that meet their initial screening criteria. Therefore, in this stage, Web sites must provide users with just enough information about a product and allow them to easily shortlist products they like for further examination.
3. *Dig in: drill into each product*—in this stage, once customers consider a product worthy of consideration, they drill into the details to determine whether the product meets their criteria. Therefore, Web sites must provide customers with useful product-specific content that describes a product in detail.
4. *Validate*: find out what people are saying—in this stage, when customers are close to a purchase decision, they look for outside validation from other customers. Therefore, Web sites can provide ratings, reviews and other such validation features to help customers during this stage.
5. *Try*: see what it is really like—in this stage, customers often want to try a product before they commit to help ensure it fits their habits, lifestyle, or the way they work. Therefore, Web sites should make it easy for customers to try products before purchasing or to return products once purchased.
6. *Buy*: in this stage, customers finally purchase the product they were evaluating. Web sites must make it easy and efficient for customers to make the purchase.

Variations of this funnel-like model have been evaluated with and applied to various paradigms including tourism [5], online keyword advertising [3], information-seeking [6], and cosmetics [7]. We posited that it can also be readily and comprehensively applied to health research recruitment, and the model has high potential for designing online research recruitment tools—such as UMHealthResearch—that could be used by multiple research study teams.

## Methods

To adapt Margolis’ version of the buying funnel for our needs, we first gathered user feedback on the previous version of UMHealthResearch. We then created the Digital Health Research Participation Funnel based on Margolis’ buying funnel and our user feedback. Finally, we updated UMHealthResearch using our new model as a design tool to create a more active health research volunteer experience.

### User Feedback and Creation of the Health Research Participation Funnel

**Fig. 1:**
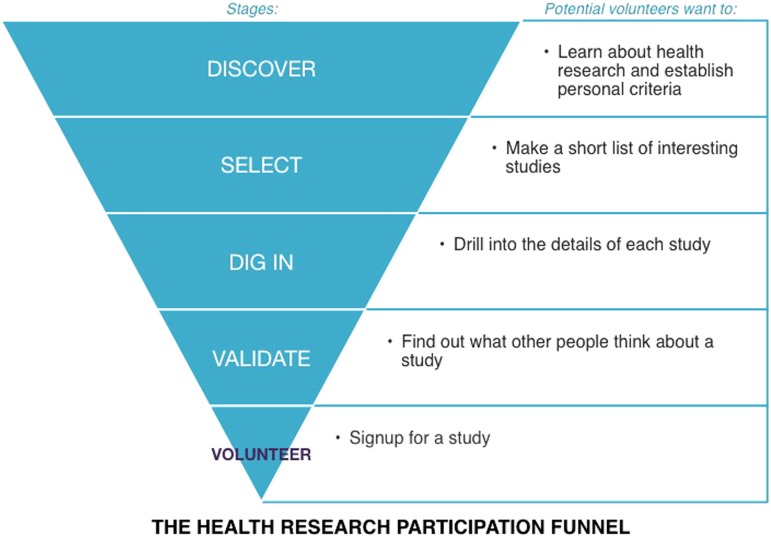
Stages of the Health Research Participation Funnel mapped to what potential volunteers want from each stage.

First, we conducted a user research study where we sought feedback from our volunteers through various channels, including in-person interviews, surveys, on-site feedback, and ethnographic studies. We also mined our customer service data to assess the kinds of questions our volunteers were asking. We then synthesized this feedback into broader questions. As a design team, we wanted to be sure to answer these broad questions that users had while searching for a study in which to participate. Thus, we mapped those broad questions to the stages of the buying funnel to create our new Digital Health Research Participation Funnel (see Fig. 1). The Digital Health Research Participation Funnel allowed us to determine how and where to address user needs and concerns. The broad questions that potential volunteers had when interacting with the previous version of UMHealthResearch can be found in Table 1.

**Table 1:** Stages of the Health Research Participation Funnel, what volunteers want in each stage, what recruitment websites must do to address these wants, and improvements made to UMHealthResearch based on these insights

	Potential volunteers want to: 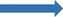	So recruitment Web sites must: 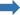	Therefore we made the following improvements:
1. Discover	*Know broad contextual information*: · What is health research? · How is health research carried out? · What are the benefits of research? · Is health research safe? · Can I trust this recruitment Web site?	Provide broad education about health research and debunk myths about participating in research	Created a home page that provides broad education about various facets of participating in health research for first-time users of UMHealthResearch through both video and text: · *Process*: What health research is and how it works · *Eligibility*: Who can participate in health research · *Need*: Why people should participate in health research · *Safety*: Whether health research is safe
	*Know broad procedural information*: · Do I have to be a patient at the University of Michigan hospitals to participate in a study? · Do I need a referral from my doctor to participate in a study?	Establish and maintain trust with users	Designed the Web site to be consistent with the University of Michigan brand in look and feel
	*Make personal preference decisions*: · What kinds of studies would I participate in? · Where am I willing to travel to? · How much time am I willing to spend on this? · How much compensation am I seeking?	Allow volunteers to choose the type of studies they want to participate in upfront	Created a way for users to search the type of studies they wanted to participate in on the home page and created a way for them to browse some popular categories of studies
2. Select	*Narrow down studies that look promising*: · Should I spend time looking into this study? · Does this study hold promise? · Am I eligible to participate? · How can I make my list of studies shorter?	Present a list of studies that fit volunteers’ criteria	Designed an easy-to-scan list of study search results based on the selected criteria
		Present information that is important to volunteers in the search results list so they can easily determine whether they want to know more about the study	Designed search results to contain key information (eg, study title, short paragraph describing study goals in lay language, gender, and age eligibility criteria)
		Allow volunteers to adjust their criteria after viewing the search result list	Created a way for volunteers to refine their search options after their initial search
3. Dig In	*Drill into the details of each study*: · What is this study about? · What is it trying to achieve? · What would I need to do? · How time-consuming is the study? · Does it offer compensation? · What if I do not want to participate anymore? · Will participation or non-participation affect my care at the University of Michigan hospitals?	Describe a particular study in detail to answer as many potential participant questions as possible	Organized the study detail page by the types of questions volunteers asked: · What is this study about? · Who can participate? · What is involved? · Is compensation provided?
			Made clear on the study details page that volunteers can always opt out of the study and they are never obligated to participate or continue participating
			Provided the research team’s contact information so volunteers can contact them for further questions
4. Validate	*Find out what others think of each study*: · How many volunteers are a part of this study? · How was their experience and was it painful? · Were the researchers nice?	Provide social validation	Created a way to display the number of people who have already shown interest in a study on the study details page
		Provide study reviews (*not feasible*)	We could not accommodate volunteers’ desire for study reviews because of ethical and privacy concerns
5. Volunteer	*Sign up for the study*: · What are my next steps? · Who should I contact for further information?	Provide an easy, actionable way for volunteers to sign up for studies and make the next steps clear	Created an “I am interested” button for volunteers to click. This button leads volunteers through a prescreening questionnaire to determine basic eligibility. If they are eligible, it then connects them to the appropriate research team
		Provide recommendations for other studies they could be a part of	Provided a list of similar studies they may be interested in based on the study they signed up for

In addition to making the new model more specific to health research participation, 2 other major differences emerged between the new Digital Health Research Participation Funnel and Margolis’ buying funnel. Specifically, we removed the “Try” stage, as this was not applicable to participating in health research through a digital tool. In addition, we replaced the “Buying” stage with “Showing Interest in a Study.” Thus, the final Digital Health Research Participation Funnel consists of 5 stages. In the “Discover” stage, potential volunteers want to learn broad contextual and procedural information about health research and they must establish their own personal criteria for the types of research they are willing to participate in. In the “Select” stage, volunteers narrow down the studies they are interested in so they have a short list of options. In the “Dig In” stage, volunteers drill into the specific details of each study of interest to ensure it meets their personal criteria. In the “Validate” stage, potential volunteers want to know what others who have participated thought about the study. Finally, as mentioned above, volunteers actively show interest in a study that they want to participate in and want to know what the next steps are.

### Design Insights Based on the Digital Health Research Participation Funnel

Creating the Digital Health Research Participation Funnel helped us understand potential volunteers’ journeys and thoughts while looking for research studies. In turn, this led to design insights for research recruitment Web sites that mapped onto the Digital Health Research Participation Funnel (see Table 1). Specifically, it is critical for recruitment Web sites to provide sufficient education about research participation, gain user trust, and provide volunteers with an easy way to search for studies when they are in the “Discover” stage. As volunteers move to the “Select” stage, they must be able to easily see relevant information for studies that fit their initial criteria to help them narrow down their options. When volunteers want to “Dig In” to study details, the Web site must quickly show them all the information they need to know in an organized fashion. For volunteers who want to learn what others think of the study, Web sites can create a method for social validation. Finally, when people are ready to volunteer, it should be quick and easy for them to show interest in the study they like and they should have a clear picture of what they need to do next. It is also ideal for the recruitment tool to connect the volunteer with other potential studies of interest to capitalize on their involvement and motivation. After determining these web design insights based on the user feedback and new model, we were able to use these insights to identify problems we needed to address to improve UMHealthResearch and create a more positive and active user experience.

### Improvements Made to UMHealthResearch based on the Digital Health Research Participation Funnel

**Fig. 2:**
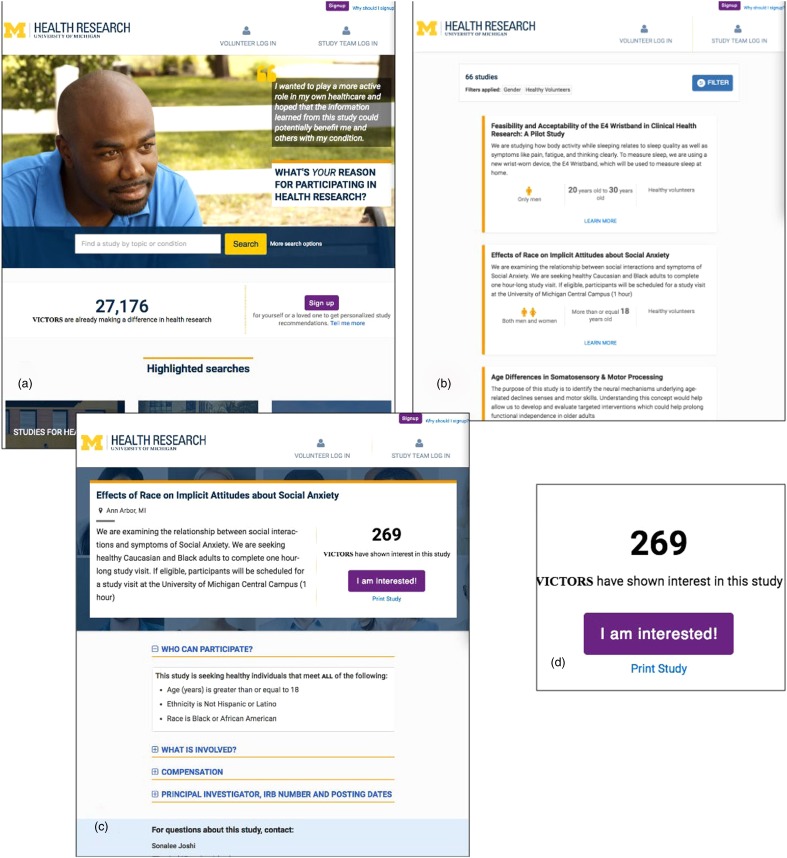
Screenshots from UMHealthResearch: (*a*) home page corresponding to the “Discover” stage; (*b*) study results list corresponding to the “Select” stage; (*c*) a page showing the details of a specific study corresponding to the “Dig In” stage; and (*d*) the number of people already interested in a study corresponding to the “Validate” stage and the “I am interested!” button corresponding to the “Volunteer” stage.

We addressed each insight garnered from the Digital Health Research Participation Funnel through one or more improvements to the previous version of UMHealthResearch (see Table 1). To help volunteers in the “Discover” stage, we ensured that the home page provided sufficient education on Digital health research through multiple modalities. For those in the “Select” stage, we created an easy-to-use search function with easy-to-read and detailed search results. To help people “Dig In,” we ensured that the study detail page answered as many questions as possible; thus, potential volunteers would be fully informed when making participation decisions. For those who seek social validation, we display the number of people who have already shown interest in a particular study. Finally, for those who were ready to volunteer, we created an easy-to-use action button, where volunteers click “I am interested!” to sign up for a study. Examples of these improvements are shown in Fig. 2.

## Results

**Fig. 3:**
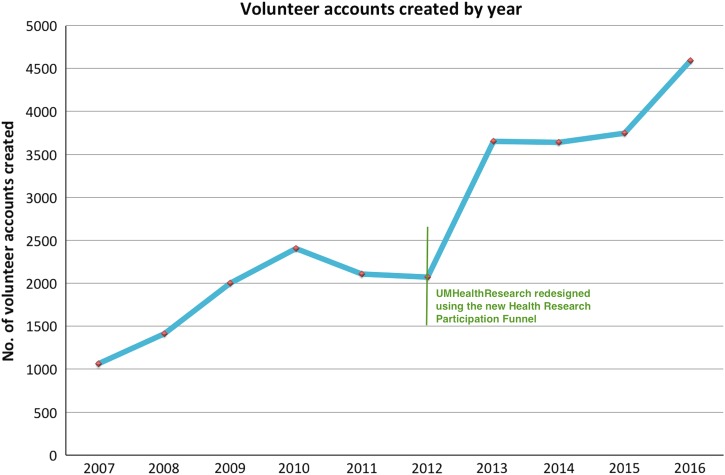
Volunteer accounts created by year from the beginning of UMHealthResearch’s inception. This includes the timeframe before the buying funnel redesign was implemented and after.

To determine how our improvements to UMHealthResearch based on the Digital Health Research Participation Funnel influenced the performance of the recruitment tool, we examined the number of volunteer accounts created before and after our redesign. As can be seen from Fig. 3, there has been a notable increase in the number of accounts created after our redesign. In the years before the redesign (2007–2012), an average of 1844 new accounts were created every year. In the completed years after the redesign (2013–2016) the average improved to 3906 accounts and more than doubled with an increase of 111%. Since the redesign, volunteers in UMHealthResearch have shown interest in a research study over 120,000 times.

**Fig. 4:**
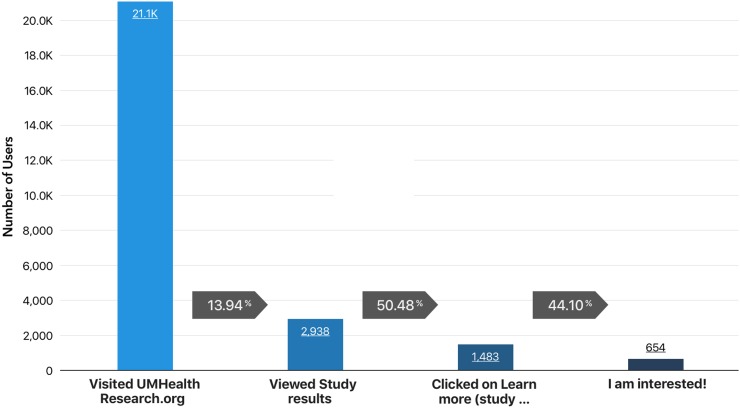
UMHealthResearch user action data through different stages in the time period between October 1, 2016 and January 27, 2017. Roughly translated, these numbers act as the metrics to measure success at each stage of the buying funnel model as applied to UMHealthResearch.

Beginning in October 2016, we started tracking conversion rates at each stage more closely using an event tracking and analytics platform (Heap analytics). In the date range between October 2016 and January 2017, 14% performed a search for research studies or browsed studies by popular topics out of all the people that visited UMHealthResearch, including researchers (to access their administrative portals) and volunteers. Out of this population, about 50% of users looked at a study in detail in the same session. Of those users, about 44% ended up showing interest in a study in that session (Fig. 4). These conversion rates allow us to measure success at each stage of the Digital Health Research Participation Funnel.

## Conclusion

In the previous version of UMHealthResearch, the focus was on volunteers registering, at which point they had to wait for a potential future match—a *passive* method. After 2013, we focused our design on enabling volunteers to sign up actively for studies. Creating the Digital Health Research Participation Funnel based on consumer engagement principles enabled us to redesign our recruitment tool to allow for a more *active* user experience. The results of preassessment and postassessment suggest that the focus on user experience improved the performance of UMHealthResearch. As UMHealthResearch grows to support more volunteers, we plan to continue evolving the recruitment tool to fit their needs based on more user feedback, quantitative data, and the Digital Health Research Participation Funnel. Specifically, we continue to focus on increasing conversion rates for each of our stages, or “plugging the leaks in the funnel.” Some new initiatives that are currently being considered include: (1) helping researchers craft postings in a lay-friendly language that is understandable by our volunteers; and (2) encouraging researchers to post more studies in the system to provide volunteers with more choices.

It must be acknowledged that we did not employ a randomized design, and thus we cannot assert unequivocally that the redesign is the causal influence that doubled registration rates. Preassessments and postassessments are subject to the confounds of other changes occurring that might be influential. However, it should be noted that there were no other major initiatives related to recruitment or UMHealthResearch.org at this time. Further, the dramatic and sustained increase after the redesign supports the hypothesis that the shift to active user engagement leads to increased performance. This strategy is likely generalizable and warrants further application in web-based platforms that support recruitment to health-related research.
